# O5.7. RISK OF DIABETIC COMPLICATIONS AND SUBSEQUENT MORTALITY AMONG INDIVIDUALS WITH SCHIZOPHRENIA AND DIABETES MELLITUS: A NATIONWIDE POPULATION-BASED REGISTER STUDY

**DOI:** 10.1093/schbul/sby015.221

**Published:** 2018-04-01

**Authors:** Anita Tønder Nielsen, Mogens Vestergaard, Trine Munk-Olsen, Jette Kolding Kristensen, Thomas Munk Laursen

**Affiliations:** 1 The National Centre for Register-based Research, Aarhus University; 2 Institute of General Medical Practice, Aarhus University; 3 National Centre for Register-Based Research, Aarhus University, The Lundbeck Foundation Initiative for Integrative Psychiatric Research (iPSYCH); 4 Research Unit for General Practice, Aalborg University; 5 Aarhus University, The Lundbeck Foundation Initiative for Integrative Psychiatric Research (iPSYCH)

## Abstract

**Background:**

Schizophrenia constitutes a high risk of morbidity and mortality from physical illness. Individuals with comorbid schizophrenia and diabetes mellitus have been found to have a three- to four-fold higher rate of death than the general population, which may be explained by a higher rate of diabetic complications. We aimed to study incidence of diabetic complications diagnosed in hospitals following a diabetes diagnosis and subsequent mortality in individuals with schizophrenia compared to individuals without.

**Methods:**

The entire Danish population was followed in 1997–2016 using population-based registries. Incident diabetes was defined as prescription redemptions of insulin or oral antidiabetic drugs, ICD-10 diagnoses E10 or E11 related to hospital contacts, whichever came first. Diabetic complications were separated into macrovascular complications (coronary heart disease, peripheral artery disease, stroke+TCI, heart failure, myocardial infraction, foot ulcer) and microvascular complications (retinopathy, neuropathy, nephropathy).

Cox regression was used to estimate incidence rate ratios (IRR) and mortality rate ratios (MRR) and all estimates were adjusted for age and calendar time.

**Results:**

The incidence rate of macrovascular complications was similar in individuals with schizophrenia and in those without; IRR=1.09 (95% CI: 0.96–1.23) for females and IRR=0.91 (95% CI: 0.83–1.01) for males. For foot ulcer the incidence rate was higher in females with schizophrenia than in females without; IRR=1.79 (95% CI: 1.12–2.85), p=0.015 and for heart failure the incidence rate was higher in males with schizophrenia than in males without; IRR=1.52 (95% CI: 1.21–1.91), p<0.000. The incidence rate for microvascular complications was similar for females with and without schizophrenia; IRR=0.88 (95% CI: 0.75–1.04), but lower in males with schizophrenia than in males without schizophrenia; IRR=0.79 (95% CI: 0.69–0.89), P<0.001.

The mortality rate following a diagnosis of a macrovascular complication was higher in individuals with schizophrenia than those without; MRR=2.17 (95% CI: 1.84–2.56) for females and MRR=2.40 (95% CI: 2.06–2.80) for males. For microvascular complications the subsequent mortality rate was also higher in individuals with schizophrenia than in those without; MRR=2.17 (95% CI: 1.67–2.80) for females and MRR=2.30 (95% CI: 1.90–2.79) for males. P<0.001 for all estimates. The Mortality rates for every single complication showed similar estimates.

**Discussion:**

Unexpectedly, we found individuals with comorbid schizophrenia and diabetes mellitus to have a similar or lower rate of diabetic complications diagnosed in hospitals compared to individuals with diabetes mellitus only. However, we still found an excess mortality following a diagnosis of a diabetic complication among individuals with schizophrenia. These results may indicate that individuals are not even seen in hospitals with their diabetic complications and hence indicate an increased need for improved somatic care of individuals with schizophrenia if the burden of diabetes mellitus morbidity and mortality should be reduced.

